# Need for Increased Awareness of International Male Circumcision Variations and Associated Complications: A Contemporary Review

**DOI:** 10.7759/cureus.24507

**Published:** 2022-04-26

**Authors:** Makayla Gologram, Ryan Margolin, Christine M Lomiguen

**Affiliations:** 1 Preclinical Medicine, Lake Erie College of Osteopathic Medicine, Erie, USA; 2 Obstetrics and Gynecology, Monmouth Medical Center, Long Branch, USA; 3 Pathology, Lake Erie College of Osteopathic Medicine, Erie, USA; 4 Family Medicine, Millcreek Community Hospital, Erie, USA

**Keywords:** circumcision, complication, urology, surgery, public health, international medicine, history taking

## Abstract

Male circumcision is a prevalent, straightforward procedure. Cultural, religious, and/or social factors are the main determinants in the decision to undergo circumcision. The method of circumcision and age of the patient at the time of circumcision varies, dependent on the deciding determinant: cultural or religious tradition, personal hygiene, preventive health, or medical need. While circumcision is a relatively simple operation for a trained medical professional with low rates of adverse events, the safety of the operation varies when performed by non-medically trained (traditional) practitioners. This review aims to inform physicians of international circumcision variations and associated complications and to provide history-taking considerations during the review of the genitourinary system. The review revealed a wide variety in 1) training of practitioners performing circumcisions, 2) methods of circumcision, and 3) sterility during the procedure contributing to differing rates of complications. Findings suggest circumcisions should not be viewed equally, and greater emphasis should be placed on genitourinary history, especially circumcision, with patients from areas where traditional circumcisions are prevalent.

## Introduction and background

Male circumcision is one of the most common surgical procedures in the world [1-2]. Roughly 38% of the global population is circumcised [3]. The procedure consists of the removal of the foreskin from the glans of the penis [1]. In the United States (USA), many male neonates are circumcised for health benefits, such as decreased urinary tract infections, decreased rate of penile cancer, and decreased risk of contracting Human Immunodeficiency Virus (HIV) [4]. Additionally, religion is one of the primary reasons for circumcisions [5]. Virtually all males worldwide of the Jewish and Muslim faith are circumcised; therefore, Middle Eastern countries with a primarily Muslim or Jewish population have extremely high rates of circumcision (>90%) [3-4]. Moreover, cultural traditions are a determinant for circumcision; many African cultures perform circumcision as a male rite of passage between the ages of five and sixteen years, depending upon the region and culture [3-4]. In the Philippines, where approximately 90% of males are circumcised, sociocultural causes are the driving force behind circumcision; boys are often between 10 and 14 years old when circumcision occurs, whereas Muslims believe it must be completed sometime between birth and puberty [5]. 

The procedure can be performed by (1) a trained medical professional, such as a physician and nurse, or by (2) religious or cultural practitioners with varying degrees of formal healthcare education, broadly referred to as traditional practitioners [4,6]. Traditional practitioners can be religious figures, such as a Jewish Mohel, trained in performing circumcisions to religious heads of a group who have learned to circumcise from performing the procedure [5]. Traditional circumcisions occur in a variety of settings from the patient's home, as is tradition for Jewish circumcisions, to the town square in rural locations [4,6]. As such, the level of sterility in which these procedures take place can vary widely [7]. 

The vast and diverse practices of circumcision warrant discussion of its safety and efficacy. With global practice variations by both surgeons and traditional practitioners, complications of each procedure consequently vary. This review aims to inform physicians of these variations when performing genitourinary exams on international patients who may not have had the usual neonatal circumcision done by a physician. Understanding disparities in the practice of circumcisions can aid physicians while discussing genitourinary health and treatment of potential long-term adverse events.

## Review

Methods

A search was conducted of the National Library of Medicine’s MEDLINE/PubMed and Google Scholar databases, with the objective of identifying all articles published in the English language with topics of “circumcision complications” or “circumcision culture” in conjunction with “traditional practitioner”. The reference lists of all articles identified by this search strategy were reviewed and all pertinent literature was retrieved, which was analyzed to identify any potential additional manuscripts. All data were accessed between March and July, 2020. The initial search yielded 25 articles for possible inclusion due to their relevance and incorporation of either varied circumcision practices, complications after circumcision, or practice by non-medical providers. This search yielded a total of 14 studies/reports, which were assessed and incorporated into this review, organizing sources of variations in international circumcision practices (Table 1).

**Table 1:** Summary of circumcision complications, location, and age. *: The study looked at only patients who had iatrogenic phimosis as a complication ^: The study looked at only patients who had meatal stenosis as a complication

	Author	Reference Type	Country	Age range	Practice Variations	Complications
1	WHO, 2008 [5]	Report	Multiple	Various	Lack of surgical instruments, retention of parts of the foreskin (non-hospital)	Excessive bleeding, hematoma, sepsis, unsatisfactory cosmetic effect, lacerations, injury to the glans, glanular amputation, infection, death
2	Weiss et al., 2010 [7]	Review	Multiple	Neonatal - childhood	Clamp method by medical provider (hospital), free hand by traditional practitioner (non-hospital)	Infection, urethral laceration, bleeding, meatal stenosis, incomplete circumcision, amputation of glans, foreskin adhesions, subcutaneous cysts, hematoma, inflammation
3	Abdulwahab-Ahmed et al., 2013 [8]	Review	Various	Various	Dorsal slit, clamp, and sleeve methods by physician and traditional practitioner (hospital and non-hospital)	Bleeding, concealed penis, phimosis, skin bridge, infection, urinary retention, fistula, necrosis, iatrogenic hypospadias, meatits, cyst, impotence
4	Krill et al., 2011 [9]	Review	Multiple	Various	Dorsal slit, clamp, and sleeve methods by physician (hospital)	Bleeding, pain, inadequate skin removal, infection, iatrogenic hypospadias, glanular necrosis, glanular amputation
5	Heras et al., 2018 [10]	Retrospective	United States	Neonatal	Clamp method by physician (hospital)	Bleeding
6	Tuncer et al., 2017 [11]	Retrospective	Turkey	Neonatal -18	Clamp method, and sleeve technique with thermocautery by physician (hospital)	Hemorrhage, infection, phimosis buried/trapped penis, meatitis, scrotal injury
7	Hung et al., 2019 [12]	Retrospective	United States	<5yo	Clamp method, sleeve technique, and dorsal slit by physician (hospital)	Hemorrhage, infection, non healing wound, reoperation
8	Ceylan et al., 2007 [13]	Review	Turkey	Various	Clamp, sleeve and dorsal slit by physician and traditional practitioner(hospital and non-hospital)	Bleeding, infection, glans amputation, urethral fistula, iatrogenic hypospadia, meatal, stenosis, preputio-glandular fusion
9	Bailey et al. 2008 [14]	Prospective	Kenya	Mean= 14.1	Medical and traditional circumcisions (hospital and non-hospital)	Infection, inflammation, hemorrhage, lacerations, meatal ulcers, meatal stenosis, necrosis, amputation, death
10	Osuigwe et al., 2004 [15]	Prospective	Nigeria	Neonatal	Clamp method by physician (hospital) and traditional method by traditional practitioner (varied hospital settings)	Bleeding, incomplete circumcision, urethral fistula, meatal stenosis, amputation of penile shaft
11	Akyuz & Cam, 2020 [16]	Case-controlled	Turkey	1-8 years old	Sleeve technique with thermocautery by physician (hospital)	Iatrogenic phimosis*
12	Saeedi et al., 2017 [17]	Cross-sectional	Iran	<6 months	N/A	Meatal stenosis^, pain
13	Bazmamoun et al., 2008 [18]	Review	Multiple	Various	Sleeve method by physician (hospital)	Bleeding, infection, incomplete circumcision, adhesions, meatal stenosis, skin bridges,
14	Okeke et al., 2006 [19]	Cross-sectional	Nigeria	8 days to 13 months	Physician, non-physician, medical personnel (hospital), and traditional practitioners (non-hospital)	Redundant foreskin, excessive loss of foreskin, skin bridges, amputation of glans, buried penis, hemorrhage

Discussion

Variations in Practice

**Figure 1:**
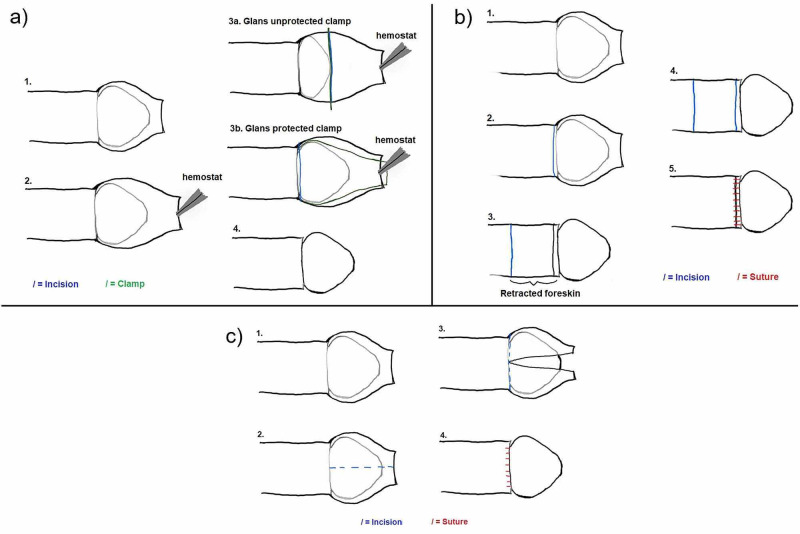
Methods of Circumcision (a) The clamp method: The foreskin is separated from the glans, and then a hemostat is used to extend the foreskin. Different clamps can be used, either glans unprotected (e.g. Mogen clamp; 3a) or glans protecting clamp (e.g.Plastibell clamp; 3b) to divide the foreskin that will be excised. (b) The sleeve method: The foreskin is retracted, edges marked, and incisions made to remove the foreskin intact. (c) The dorsal slit method: The foreskin is separated from the glans, an incision is made longitudinally along the foreskin, before making a circumferential incision to remove the foreskin. Original illustrations by Ryan Margolin.

The methods used for circumcision vary by practitioner and location [4]. Clinic-based procedures comprise (1) the sleeve removal, which requires the most surgical training but produces the cleanest result, (2) the dorsal slit method, and (3) the clamp method, done with a variety of different clamps based on the practitioner’s preference (Figure 1) [4,8-9]. These procedures can use electrocautery and clamps specifically designed to protect and/or visualize the glans, both of which help provide immediate hemostasis [10,20]. Outside of the clinic, traditional practitioners tend to use a modified version of the clamp method with items such as string or a metal guard; others perform the circumcision “freehand”, or unguided, with a razor blade, penknife, broken shells, or a different sharp instrument [4]. Practitioners can also vary in the type of anesthesia they use. Usually, medically-trained clinicians use general anesthesia or a penile block on circumcisions performed after the neonatal period, whereas traditional practitioners may use local anesthesia or no anesthesia [21]. Studies have reported a decreased rate of complications when general anesthesia is used versus local anesthesia [11].

Complications

There are numerous possible complications that can occur due to circumcision, including, but not limited to: bleeding, infection, pain, incomplete removal of the foreskin, amputation of glans, glans necrosis, skin bridge formation, meatal stenosis, and death (Table 2) [4,7-9,11,22]. The rates of these complications vary based on patient age, practitioner training, and method of circumcision (Table 3). Concerning age, an increase in complications occurs in patients with circumcision after the neonatal period and into adolescence (6%) as opposed to neonates (1.5%), in part due to increased bleeding in non-neonates, believed to be a result of increased vascularity of the foreskin after the neonatal period [7,9,12]. In many cultures in which traditional practitioners and untrained volunteers perform circumcisions, the procedures occur in adolescent patients posing greater risks for complications than when done by medical providers [3-4]

**Table 2:** Summary of possible complications of circumcisions, acute and long-term.

Acute	Long-Term
Bleeding	Incomplete removal of foreskin
Infection	Iatrogenic phimosis
Pain	Skin bridge formation
Delayed wound healing	Meatal stenosis
Amputation of glans	Psychological trauma
Glans necrosis	Fistula formation
Death	Iatrogenic hypospadia

**Table 3:** Factors contributing to increased risk of complications during circumcision.

Patient Based	Practitioner Based
Advanced age of patient	Sterility of location and instruments
Contraindication for circumcision (e.g. hypospadia)	Training and medical knowledge of practitioner
	Mass circumcision

The setting of the procedure can vary from the sterility of an operating room to unsterile locations typically utilized for daily activities, such as a classroom, town square, or one’s home [4,6-7,23]. Unsterile environments, equipment, and practices coupled with the varying skill of traditional practitioners have been shown to increase the complication rate [7,13-14,23]. Complication rates from developing countries, however, are difficult to ascertain due to a lack of data from inconsistent or incomplete record taking [4]. 

Pain: Post-operative pain is the most frequent side effect, especially when performed by non-medical practitioners. Studies about circumcisions performed without a doctor and not in the medical setting have reports of pain by patients as high as 64.5% [6]. The most appropriate time to perform circumcision with minimal pain is within the first week after birth, but long-term pain after infant surgery is still possible with patients identifying painful urination years later secondary to other complications such as having a skin bridge or meatal stenosis [17]. Pain levels in patients receiving circumcision in the hospital setting and by a physician trained for the procedure are considerably lower because of the use of anesthesia and surgical equipment, knowledge, and typically younger age of the patient [24].

Bleeding: Bleeding is the second-most common complication seen in circumcisions [4,7,9,21]. Of the reviewed literature, 12 of 14 studies listed bleeding or hemorrhage as a complication of circumcision. The true number of bleeding complications is difficult to determine due to differences in the qualification and quantification of complications [7]. Some studies classify bleeding that can be stopped easily with pressure as not to count as a complication, whereas others report any bleeding as a complication [4,7]. The reported rates of bleeding can vary drastically in studies, ranging from 0.1% to 35% of cases [8]. In a review of complications in Nigeria of circumcisions performed by doctors, nurses, and traditional practitioners, bleeding was found in 7.8% of the cases [15]. In a study comparing complication rates between circumcisions performed outside the hospital by traditional practitioners versus licensed surgeons, the rate of bleeding was 23.8% in traditional practitioners compared to only 1.2% by licensed surgeons [23]. In a large statewide study from California, USA, the rate of hemorrhage among neonates was 0.32% while the rate in non-neonate patients was 1.55% [12]. A review of two community hospitals in New York, USA, found that 41 of 1064 (3.9%) neonates had a hemorrhage, and only three patients (0.3% of all participants) required sutures to stop the bleeding [10].

Infection: Infection after circumcision is a common postoperative complication, especially when performed by a traditional provider and if proper surgical equipment, sterilization, and post-surgical wound care were lacking [4,14]. Most infections are considered early complications and easily treatable in the hospital setting with antibiotics, but some infections, although very rare, can pose the threat of necrotizing fasciitis by polymicrobial sources most commonly, Group A *Streptococcus* and *Staphylococcus aureus *species, requiring surgical debridement [9]. Postoperative infections can be seen in circumcisions performed by physicians and by non-medical practitioners, but higher rates of infections are seen in those with no formal medical education (14%) and when compared to those performed in a hospital setting (6.6%) [7,11].

Incomplete removal of foreskin, delayed phimosis: Incomplete removal of the foreskin may be underreported, as it is not always medically relevant unless it causes phimosis. Unless a patient goes for a revision because of an undesirable appearance or excess tissue causing phimosis, there may be no record that circumcision was incomplete [9,22]. Some studies also do not consider incomplete removal of the foreskin or undesirable appearance to be a true medical issue, and thus, it may not be included in the complication rates [7]. However, in studies looking at revision circumcisions, incomplete removal of foreskin leading to an undesirable appearance or iatrogenic phimosis are common causes of revision circumcision [22]. It was found that redundant foreskin constituted 53% of complications in a study looking at Nigerian circumcisions done by physicians, nurses, and traditional practitioners. More complications occurred when nurses performed the procedure [19]. Another study looking at 700 boys circumcised in a hospital over a five-day period found that 2.1% of patients had phimosis due to incomplete foreskin removal [7]. Less than one percent (0.36%) of patients had to undergo circumcision revision due to phimosis in a study looking at patients who returned to the hospital [16]. One study found that six out of 21 (28.6%) patients who experienced complications had secondary phimosis due to circumcision [11]. Another study looking at late complications treated at Massachusetts General Hospital, US, found that 5.8% of cases were due to iatrogenic phimosis. This study found that 231 (40.1%) of the revision circumcisions were due to incomplete removal of the foreskin during the initial circumcision [22]. When comparing traditional practitioners to physicians performing circumcisions, one study found that 11.8% of males circumcised by traditional practitioners had incomplete circumcisions, while no physician-performed circumcision resulted in incomplete removal [23]. Another study looking at doctors, nurses, and traditional practitioners found a rate of 9.9% incomplete circumcisions with 43% occurring from traditional methods [24].

Amputation of the glans: Among acute adverse events, amputation of the glans is, fortunately, rare. In a study conducted in Nigeria, the majority of patients presenting to a welfare clinic had a circumcision performed by nurses with glans amputation only occurring at a rate of 1.5% [19]. This serious complication requires reanastomosis of the amputated tissue and can be seen in procedures that fail to protect the glans, such as those using the Mogen clamp, the sleeve method, or the dorsal slit method [9].

Glans necrosis: Although a rare complication, higher rates of glans necrosis can be seen in specific techniques, and management depends on the severity of the necrotic tissue [9]. Improper use of circumcision-specific surgical equipment can increase the risk of glans necrosis and is the most reported cause of the event [8,9]. The Gomco circumcision has reported glans necrosis resulting from a cautery injury and the Plastibell ring has caused necrosis when it is inappropriately sized [8,9]. 

Skin bridge: Skin bridges can occur as a post-circumcision complication due to thick tissue adhesions that form between the glans and the shaft of the penis. Skin bridges, if left untreated, can cause issues such as tethering of the penis resulting in an abnormal curvature during an erection and it can affect one’s hygiene and ability to keep the genitals clean by allowing for an area in which smegma can accumulate [8-9]. These skin bridges require surgical removal unless they are thin [9]. In a study from Massachusetts General Hospital, USA, 27.8% of the revisions were to fix skin bridges [21]. When comparing traditional practitioners' rates of adhesions compared to physicians’ rates, 3.68% of cases were complicated by adhesions compared to 0.1% of cases respectively [23]. 

Meatal stenosis: Meatal stenosis is the narrowing of the urethral meatus. A normal urethra in a child younger than four years old is 3.33 millimeters in diameter with an ellipsoid shape [25]. Stenosis can be considered if the meatus becomes pinpoint, or if the urethra can not accommodate a two-millimeter in diameter foley catheter [16,18,25]. It is a complication that is usually asymptomatic until toilet training if it ever becomes symptomatic [9]. It presents as a urinary stream deviation, dysuria, narrow stream, and potentially urinary retention if severe [9, 22]. A study examining patients with complications due to circumcision found that 23% of the complications were meatal stenosis that required meatotomy [13]. Another study surveying patients with complications found similar results with 26.1% of patients requiring a meatotomy for symptomatic meatal stenosis [22]. A study looking at circumcisions in Iran found that the rate of meatal stenosis among participants was 3.3% [25]. In the study looking at doctor-, nurse-, and traditional practitioner-performed circumcisions, 3.5% of patients had meatal stenosis [15]. Meatal stenosis is a condition that requires an extensive physical exam to discover. A study of males with clinically diagnosed meatal stenosis found that 26.6% of the patients were asymptomatic and were found by chance [18].

Death: Death by circumcision has been sparsely reported in the literature. Most cases of death were indirectly caused by circumcision, but poor surgical aftercare such as dehydration and exposure to the elements were the main contributors [14]. This complication is linked to circumcisions performed by traditional practitioners and mass circumcisions [4,14]. Death in hospital-based circumcision practices occurring with a physician has not been reported.

## Conclusions

Circumcision is a widely performed procedure that carries risks that must be considered to mitigate the likelihood of occurrence. The prevalence of circumcision is unlikely to decrease due to its use in cultural and religious rituals. Considering the diverse methods, conditions, and age at which patients undergo circumcision, it is important to consider that not all circumcisions are the same. As these rituals are often performed by traditional practitioners, long-term complications such as meatal stenosis, iatrogenic phimosis, skin bridges, and undesirable appearance should be considered. Complications are not exclusive to those performed by traditional circumcisers, as they can occur when performed by licensed, trained professionals. Previous studies have concluded that due to complications and risks, circumcisions should be done by medically trained professionals in a sterile setting. However, given the geographical isolation and/or cultural implications of places where circumcisions are endemic, this is unlikely to happen everywhere. As such, when discussing the history of a patient, it is important to not only determine if they have had a circumcision but to also evoke a more detailed history such as age, method, and location of the procedure, especially in patients from areas where traditional practitioner circumcision is common. This information can help guide clinicians during the initial visit and/or for focused genitourinary complaints toward patients who may be at higher risk of complications, in order to ask the right questions, as this may not be something every patient brings up naturally. Long-term consequences such as incontinence, recurrent urinary tract infections, sexual dysfunction, impotence, and pain with genital function, should not be ignored by physicians, and exploration into causes of circumcision, especially if done by a non-medical provider, should be examined. Future studies can aim to create a standard set of questions to obtain specific patient circumcision history and can subsequently assess the success and/or failures of utilizing this tool.
